# Contemporary Role for Blue Light Cystoscopy Across the Bladder Cancer Disease Spectrum

**DOI:** 10.1007/s11934-026-01322-7

**Published:** 2026-02-14

**Authors:** Ethan Wan, Caroline Wade, Aditya Sathe, James E. Ferguson, Charles C. Peyton

**Affiliations:** 1 Heersink School of Medicine, University of Alabama at Birmingham, Birmingham, AL USA; 2 Department of Urology, University of Alabama at Birmingham, Birmingham, AL USA

**Keywords:** Blue-light cystoscopy, Bladder cancer, Photodynamic diagnosis, Hexaminolevulinate, Transurethral resection of bladder tumor

## Abstract

**Purpose of Review:**

This review aims to synthesize recent evidence and accounts evaluating the utility of blue light cystoscopy (BLC) across the disease spectrum of bladder cancer, including its diagnostic, surveillance, and post-treatment uses, as well as its outpatient applications, economic impact, patient perspectives, and future directions.

**Recent Findings:**

While initial studies on BLC suggested an advantage over traditional cystoscopy in terms of diagnostic power and recurrence rates for non-muscle invasive bladder cancer, recent studies are more equivocal on its benefits and suggest that the utilization of blue-light cystoscopy is more nuanced. Evidence is lacking on a clear superiority of blue light for all bladder cancer patients. Cost, logistics, and accuracy concerns indicate certain patients may benefit from this procedure more than others.

**Summary:**

BLC can be a valuable adjunct to white light cystoscopy in the management of bladder cancer. It provides high diagnostic sensitivity but there are questions surrounding its cost-effectiveness, positive predictive value, and its role in effective surveillance and reduction of tumor recurrence require further longitudinal and standardized investigation.

## Introduction

Bladder cancer (BC) is the 7th most common form of cancer globally, with over half of BC deaths occurring in countries with high area of deprivation indices (ADI) [1]. The most common form of BC is non-muscle-invasive bladder cancer (NMIBC), which is defined as malignancies limited to the bladder mucosa and submucosa [2]. NMIBC can progress to muscle-invasive forms and has high recurrence rates due to tumor re-implantation, field effect changes, and incomplete identification and removal [3, 4]. White light cystoscopy (WLC) is the gold standard for identifying lesions for biopsy and removal, but this modality can be prone to false negatives, especially when the lesion is flat, low grade or visually indistinct from the adjacent bladder mucosa [5, 6].

Blue light cystoscopy (BLC) was developed as an enhanced visualization technique to offer improved sensitivity over WLC, particularly for detecting flat lesions such as carcinoma in situ (CIS), which are often missed during standard cystoscopy. BLC was approved by the FDA in the US with rigid cystoscopy in 2010 and was subsequently approved for use with flexible cystoscopy in 2018, expanding its utility from the operating room to the office surveillance setting. Since then, numerous randomized trials and meta-analyses have demonstrated that BLC can improve detection rates and may also reduce tumor recurrence, improve risk stratification, and can potentially delay progression to muscle-invasive disease. This review examines the evolving role of blue light cystoscopy, outlining the latest clinical evidence, practical applications, limitations, and prospects.

## Clinical Landscape of Bladder Cancer

Urothelial carcinoma accounts for over 90% of bladder cancers and 75% are NMIBC at diagnosis [7]. The management of non-muscle invasive bladder cancer is challenging due to its biological heterogeneity, multifocality, and incomplete eradication with local resection. If left untreated, approximately 20–30% of all patients will progress and 50–70% of patients will experience a recurrence [8]. Risk stratification is based on clinical and pathological features such as tumor size, number, grade, stage, and presence of CIS.

A defining feature of NMIBC biology is the concept of field cancerization. Unlike organ-confined cancers such as renal cell carcinoma (RCC) or prostate cancer (PCa), which typically present as isolated lesions, it is hypothesized that bladder cancers arise from a premalignant urothelial field. This diffuse predisposition to tumor formation, along with residual disease from incomplete resections, can explain the multifocality, recurrence, and challenges of managing NMIBC [9].

Even with improvements in diagnostic methodology and treatments, BC imposes one of the highest lifetime treatment costs of all cancers, with per-patient costs are routinely estimated from $20,000 to over $100,000 depending on stage [10–14]. The cost burden of BC is primarily due to the need for long-term surveillance and repeat transurethral resections necessitated by incomplete resections and the inability to detect flat lesions such as CIS, increasing both direct medical expenditures and indirect costs such as loss of productivity and reduced quality of life. This diagnostic gap highlights an unmet need for improved visualization techniques that can enhance tumor detection enabling complete resections and improved risk adaptation.

### Mechanism of Blue Light for Enhanced Detection of Tumors

**Fig. 1:**
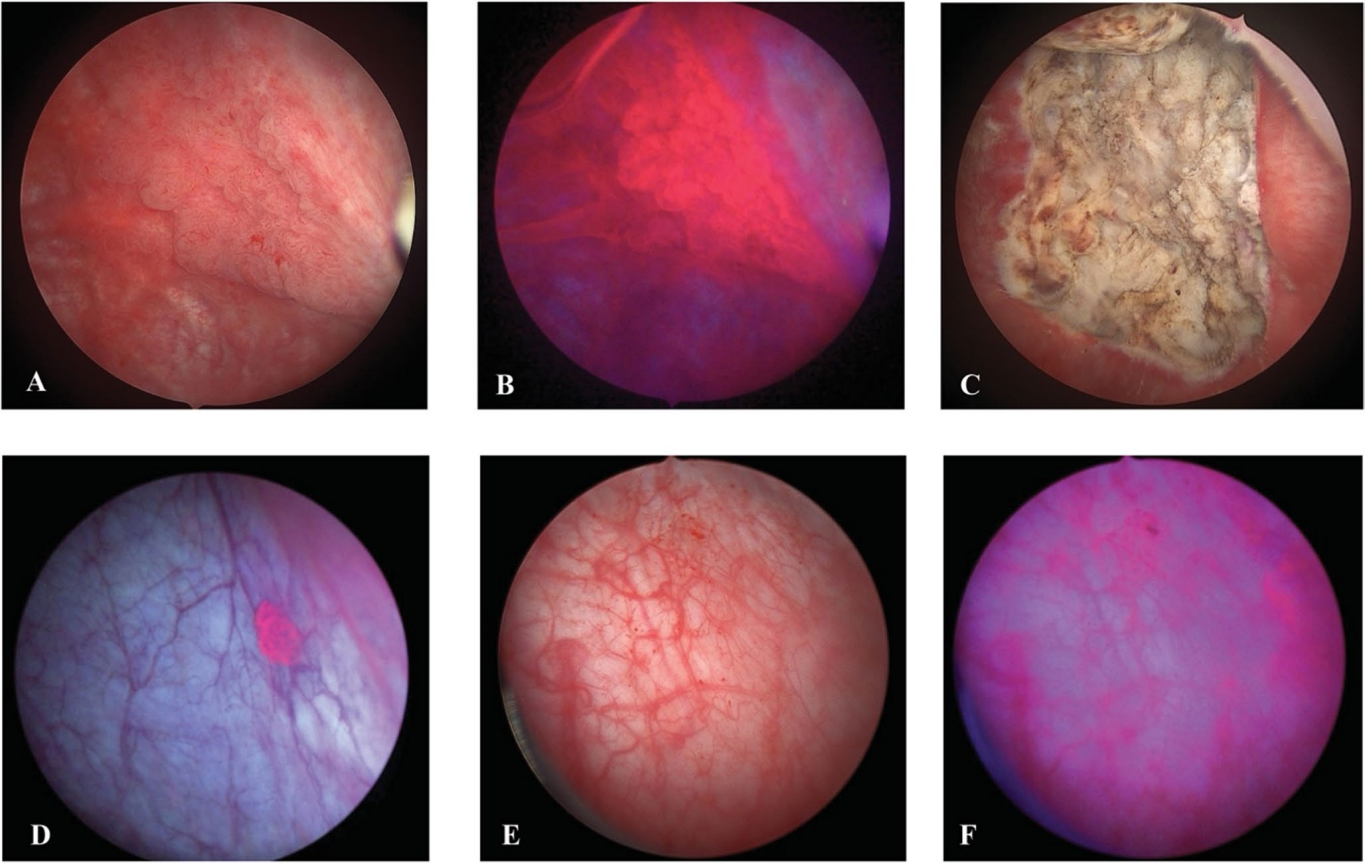
Select images of blue light and white light cystoscopy. High-grade tumor on white light cystoscopy (**A**) and blue light cystoscopy (**B**). High-grade tumor bed after complete transurethral resection under white light (**C**). Isolated papillary lesion under blue light (**D**). Bladder inflammation under white light (**E**) with corresponding blue light false positive (**F**)

BLC begins with intravesical instillation of 5-aminolevulinic acid (5-ALA), or its derivative hexyl-aminolevulinate (HAL), for at least 1 h prior to the procedure [15]. These are heme precursors that accumulate preferentially within neoplastic tissue. They are then metabolized to protoporphyrin IX, instead of completing the heme biosynthesis pathway due to the impaired ferrochelatase function of cancer cells [16, 17]. The accumulation of protoporphyrin IX causes malignant areas to emit red under blue light which provides an optical contrast against normal bladder mucosa, which appears blue or pale. Whereas white light can only show mucosa, BLC offers a visual dichotomy, which a surgeon can use to identify lesions for removal during TUR. Surgeons can alternate between white and blue light modes to monitor the resection progress intraoperatively (Fig. 1). BLC can also be performed for surveillance after initial BC resection.

The interpretation of red fluorescence requires clinical judgment because it is not always specific to cancer cells. 5-ALA or HAL uptake and protoporphyrin IX accumulation can also occur in areas of active inflammation, urothelial regeneration, or recent bladder manipulation, which may result in false positives (Fig. 1). It is therefore advised not to use BLC in patients that with gross hematuria, porphyria, a possible hypersensitivity to HAL or 5-ALA derivatives, or those who received Bacillus Calmette-Guérin (BCG) treatment within the last 90 days [18, 19]. Additionally, it is recommended that the bladder is washed out multiple times with saline prior to visualizing the mucosa with blue light to reduce the likelihood of false positives.

## Methods

We conducted a narrative review of the literature using PubMed, EMBASE, Web of Science, Academic Search Premier, Google Scholar, and SCOPUS for articles published between 2005 and 2025, prioritizing those between 2020 and 2025, with the keywords “blue-light cystoscopy OR blue light cystoscopy OR photodynamic diagnosis”. Reviews, research articles, case reports, clinical trials, editorial comments, and conference abstracts/presentations were considered. We found 1056 articles pertaining to the topic and 99 were selected for inclusion in this review based on relevance to the discussion.

## Results

### Non-Muscle Invasive Bladder Cancer

#### Primary BLC

The use of BLC in NMIBC is well-documented and can be useful for detection. The American Urological Association (AUA) currently moderately recommends, with Grade B evidence strength, physicians offer BLC in patients who have or are suspected of having NMIBC to increase detection and decrease recurrence [20]. Most professional guidelines recognize BLC as a useful adjunct to WLC and recommend its use if available (Table 1) [20–26]. Several recent studies emphasize that BLC can identify additional bladder cancer lesions that are not seen on standard cystoscopy [27–30].

**Table 1:** Current urological society recommendations on blue light use

Society or Source	Year	Guidelines	Recommendation Strength
American Urological Association/Society for Urologic Oncology [20]	2024	In a patient with NMIBC, a clinician should offer BLC at the time of TURBT, if available, to increase detection and decrease recurrence.	Moderate, Grade B
American Urological Association/Society for Urologic Oncology [21]	2016	In a patient with history of NMIBC with normal cystoscopy and positive cytology, a clinician should consider prostatic urethral biopsies and upper tract imaging, as well as enhanced cystoscopic techniques (BLC, when available), ureteroscopy or random bladder biopsies.	Expert Opinion
European Urological Association [22]	2024	During follow-up in patients with positive cytology and no visible tumor in the bladder, mapping biopsies or PDD-guided biopsies (if equipment is available). BLC has been shown to improve the detection of bladder cancer, especially CIS.	Strong
National Comprehensive Cancer Network [23]	2025	BLC could be helpful to detect lesions not seen on white-lightand may be used at diagnosis/TURBT and surveillance as an adjunct.	Ungraded
Japanese Urological Association [24]	2019	Recommend BLC for tumor visualization. Covered by Japanese health insurance.	Strong, 1a
Canadian Urological Association [25]	2021	When available, BLC can increase tumor detection and reduce recurrence risk.	Weak
Chinese Clinical Practice Guidelines [26]	2021	If the equipment and operators are available, BLC can be used for patients suspected of having multiple tumors, CIS or high-grade tumors, or for patients with positive urine cytology but negative ordinary cystoscopy.	Weak, LE 1a

Low-grade BC lesions can possess a papillary appearance which are easily identifiable, but flat lesions and more subtle papillary lesions can often flank the primary lesion or be present in satellite lesions elsewhere along the urothelium. These are often missed using WLC. Studies in the past 5 years measure the sensitivity of BLC to be above 90%, with BLC outperforming WLC in this metric (Table 2) [27–30]. Recent studies are consistent with prior evaluations, confirming high sensitivity of BLC to detect tumors [31, 32].

**Table 2:** Published studies in the last 5 years investigating BLC in NMIBC, excluding Meta-Analyses

Author	Year	Patients (*N*)	Study Design	Relevant Statistics
Immediate Second Look or Direct Comparison				
Taoka et al. [27]	2025	144	Nonrandomized prospective investigation of diagnostic accuracy of WLC and BLC within each patient	Sensitivity: 98.4% (BLC) vs. 95.3% (WLC) * p* = 0.281 Positive predictive value: 88.0% (BLC) vs. 92.4% (WLC) * p* = 0.311 Percent of lesions detected by BLC only: 33% vs. 3.1% for WLC * p* < 0.001
Ladi-Seyedian et al. [28]	2024	1292	Nonrandomized retrospective Cysview^®^ database containing patients who underwent BLC and WLC during TURBT from 2014–2021	Sensitivity: 96% (BLC) vs. 89% (WLC) vs. 99% (combination) * p* < 0.001 Positive predictive value: 82% (BLC) No *p*-value CIS detection rate for BLC: 93% * p* < 0.001(when compared to WLC, exact WLC detection rate unstated)
Watanabe et al. [29]	2021	83	Nonrandomized retrospective patients underwent BLC+TURBT after suspicious WLC	Sensitivity: BLC – 90.9% No *p*-value Positive predictive value: BLC – 51.3% No *p*-value
Andersson et al. [30]	2021	66	Nonrandomized prospective patients with negative WLFC but suspicious urine cytology who underwent BLFC	Percent of lesions detected by BLFC only: 48% No *p*-value
Restaging or Repeat TURBT				
McElree et al. [35]	2025	297	Nonrandomized retrospective BLC restaging in patients 3 months after 1 + induction treatments	Percent of lesions detected by BLC only: 1 treatment – 6.0% 2 treatment– 7.4% 3 + treatments – 19% No *p*-value
Chan et al. [36]	2023	101	Nonrandomized prospective two-arm study: Arm 1 – Primary BLC Arm 2 – Re-resection BLC in patients with previous WLC	31% of lesions detected on BLC re-resection after previous WLC * p* = 0.0277 3.4% of patients upstaged to MIBC on BLC re-resection * p* = 1.0 Recurrence rates after 2 years: 33.3% (Arm 1) vs. 37.5% (Arm 2) No *p*-value
Alsyouf et al. [37]	2023	115	Nonrandomized retrospective two arms: Arm 1 – BLC+TURBT followed by BLC restaging TURBT Arm 2 – WLC+TURBT followed by BLC restaging TURBT	Percent of patients with benign pathology on restaging TURBT: 47% (Arm 1) vs. 30.0% (Arm 2) * p* = 0.08 Rates of upstaging to MIBC: 3% (Arm 1) vs. 4% (Arm 2) * p* = 0.78
Lorusso et al. [38]	2022	82	Nonrandomized retrospective two arms: Arm 1 – WLC+TURBT followed by BLC reTURBT Arm 2 – BLC+TURBT followed by WLC reTURBT	Residual tumor at reTURBT: 71.4% (Arm 1) vs. 12.5% (Arm 2) * p* < 0.001 Median recurrence-free survival time: 15 months (Arm 1) vs. 32 months (Arm 2) * p* = 0.70
Morelli et al. [33]	2021	136	Nonrandomized retrospective study examining high-risk NMIBC patients who underwent a full BCG induction and had an early post-operative BLC	Sensitivity for BCG refractory tumors: 91% (BLC) vs. 41% (WLC) * p* < 0.001 Specificity for BCG refractory tumors: 75% (BLC) vs. 86% (WLC) * p* < 0.001
Surveillance and Follow-Up Detection				
Chappidi et al. [39]	2022	282	Nonrandomized retrospective analysis of patients who received BCG within 1 year prior to BLC	13% of recurrent lesions seen on BLC only No *p*-value
Lotan et al. [40]	2021	190	Nonrandomized prospective consecutive patients undergoing office-based BLFC	33% of patients with positive WLC had additional lesions detected by BLFC only No *p*-value
Recurrence and Long-Term Oncologic Outcomes				
Das et al. [41]	2023	378	Nonrandomized retrospective BLC in all patients, with data collected on previous WLC if available	Estimated recurrence rates after 2 years (%, 95% CI): BLC – 35% (29% − 40%) WLC – 48% (41% − 55%) * p* < 0.05 Recurrence risk (HR, 95% CI): BLC vs. WLC – 0.70 (0.54–0.90) * p* = 0.005
Miyake et al. [42]	2023	1578	Nonrandomized retrospective two arms: Arm 1 - Primary WLC Arm 2 - Primary BLC	10-yr bladder recurrence-free survival (HR, 95% CI): BLC vs. WLC – 0.73 (0.56–0.96) * p* = 0.020 10-yr high grade tumor recurrence-free survival (HR, 95% CI): BLC vs. WLC – 0.65 (0.46–0.91) * p* = 0.017 10-yr IBCG-defined progression-free survival (HR, 95% CI): BLC vs. WLC – 0.57 (0.33–0.98) * p* = 0.044
Nakagawa et al. [43]	2023	99	Nonrandomized retrospective two arms: Arm 1 – BLC + BCG Arm 2 – WLC + BCG	2-yr recurrence-free survival (HR, 95% CI): BLC vs. WLC – 0.41 (0.22–0.79) * p* = 0.025 2-yr progression-free survival (HR, 95% CI): BLC vs. WLC – 0.60 (0.24–1.48) * p* = 0.19 2-yr recurrence-free survival in CIS patients (HR, 95% CI) BLC vs. WLC – 0.29 (0.11–0.77) * p* = 0.039 2-yr progression-free survival in CIS patients (HR, 95% CI) BLC vs. WLC – 1.11 (0.27–4.54) * p* = 0.88
Heer et al. [44]	2022	538	Randomized prospective two arms: Arm 1 – BLC+TURBT followed by surveillance WLC Arm 2 – WLC+TURBT followed by surveillance WLC	Recurrence of bladder cancer adjusted for prespecified baseline variables (HR, 95% CI): BLC vs. WLC – 0.94 (0.69–1.28) * p* = 0.70
Gierth et al. [45]	2021	129	Randomized prospective two arms: Arm 1 – WLC+TURBT followed by immediate and maintenance chemo Arm 2 – BLC+TURBT followed by immediate chemo only	2-yr combined recurrence or death-free survival (HR, 95% CI): Arm 2 vs. Arm 1–1.29 (~ − 2.45) * p* = 0.249
Drejer et al. [46]	2020	699	Randomized prospective two arms: Arm 1 – WLC after initial TURBT Arm 2 – BLC after initial TURBT	8-month recurrence (OR, 95% CI) Arm 2 vs. Arm 1–0.67 (0.48–0.95) * p* = 0.02

Additionally, it is important to consider treatment plan modifications due to BLC. Morelli et al. reported that 16 of 136 patients in a retrospective cohort had a management change due to BLC, including 4 new BCG inductions [33]. A 2012 randomized controlled study also found that BLC changed overall management strategies more often than WLC with a higher incidence of new BCG inductions and chemotherapy (*p* < 0.001) [34]. While beneficial in isolation, this trend is complicated by BCG shortages. The rise in resource utilization must be justified by demonstrable clinical benefit and considering whether BLC-driven management changes will result in meaningful outcome improvements is essential.

#### Surveillance and Re-TURBT

Surveillance is a key component of NMIBC management. BLC has demonstrated utility in monitoring for recurrent lesions and identifying previously missed lesions on reTURBT, even in patients with previous BCG treatments (Table 2) [33, 35–40]. Recent studies show that BLC may be able to identify more lesions on post-TURBT cystoscopies compared to WLC, which aligns with a commonly cited prospective within-patient investigation that estimated BLC can detect 20% of recurrence undetectable with WLC (*p* < 0.001) [41]. However, these studies highlight a major gap in current BLC understanding: its long-term repeated utility. Only Lotan et al. included patients that underwent more than one BLC, and time-frames for studies are often unclear, so conclusions about outcomes if BLC were to be used multiple times for follow-up are not possible [40]. It will be crucial for future studies to follow patients over longer time periods and compare the utilization of BLC and WLC in multiple instances.

#### Carcinoma-In-Situ

CIS lesions are flat, high-grade and confined to the urothelium. They can be resected if identified, and thus these patients are good candidates for BLC guided TURBT. Multiple studies since the advent of BLC have proposed that BLC is more reliable than WLC for diagnosing and following-up on patients with CIS [41–44]. Notably, a Phase III prospective within-patient multicenter trial in 2007 testing BLC vs. WLC for CIS showed that WLC missed significantly more CIS lesions than BLC (*p* < 0.05) [45].

Recently, Ladi-Seyedian et al. retrospectively found that BLC sensitivity for CIS lesions was over 90%, which was significantly higher than that of WLC (*p* < 0.001) [28]. Additionally, multiple sources indicate that BLC is beneficial in patients either with positive urine cytology but negative WLC or CIS lesions [29–31, 46]. However, the value of flexible BLC (BLFC) to detect CIS is questionable according to a 2024 meta-analysis examining 10 studies and 1634 patients [47]. Although the data trended toward favoring BLC vs. WLC for CIS detection, this was not statically significant (OR 1.19, 95% CI 0.82–1.69). Therefore, a balanced, patient specific approach to the benefit of BLC in detecting CIS is encouraged.

#### Recurrence and Progression

Studies published in the last 5 years have conflicting results on whether initial BLC helps decrease future recurrence based on claimed more complete initial resections (Table 2) [36, 48–53]. One retrospective analysis of > 1500 cases reported improved 10-year recurrence-free survival (RFS) and IBCG-defined progression-free survival (PFS) in those who received initial BLC opposed to WLC (*p =* 0.020, 0.017, respectively) [49]. Another retrospective study also found increased 2-year RFS in CIS patients who received BLC vs. WLC (*p =* 0.029), albeit without a difference in 2-year PFS (*p =* 0.88) [50]. A 2022 systematic review examining 16 randomized trials over 4300 patients concluded with low certainty that BLC reduces risk of recurrence over time (*p* < 0.05), but disease recurrence and progression benefit are dependent on patient baseline risk, with higher risk patients benefitting more [54]. Veeratterapillay et al. examined 12 randomized controlled trials and saw that initial BLC had statistically significantly decreased 1- and 2-year risk of recurrence with moderate quality evidence (*p* < 0.05) [55].

Conversely, the U.K. based PHOTO trial, a frequently referenced prospective randomized trial for BLC, reported that BLC guided initial TURBT did not reduce recurrence rates and was not cost effective [51]. 538 patients were randomized to BLC guided TURBT or standard TURBT with a median follow up of 44 months. Three-year recurrence free survival rates favored WLC by an absolute difference of 3.8% (95% CI, −13.37–5.59), which was not statistically significant. Furthermore, the authors report BLC provided no evidence of difference in quality-adjusted life years (*p* = 0.444). This study should offer pause in the rapid adoption of up-front utilization of a costly technology; however, it favored intermediate risk, grade 2 tumors, and CIS was only present in 13% of specimens. Surveillance and/or further treatment was carried out using standard WLC. Therefore, the patients most likely to benefit from BLC (i.e.: CIS and high risk NMIBC) were underrepresented. In the context of multiple systematic reviews suggesting decreased recurrence rates with use of BLC, the PHOTO study results could suggest that BLC is most useful in recurrent or high-risk NMIBC as opposed to the primary setting [47–56].

### False Positives

Despite various possible benefits, one prominent drawback of BLC is its propensity to generate false positives, which is any lesion that is fluorescent under BLC without a histologically confirmed malignancy. Additionally, the viewing angle during BLC plays a critical role in image interpretation. Suboptimal angulation of the cystoscope can cause tangential illumination of the bladder wall, which may produce artifacts that mimic fluorescence. This can lead to false-positive findings, particularly along mucosal folds, at the bladder neck, or near areas of inflammation or recent instrumentation (Fig. 1). Small solitary bright dots can also be found upon examination, though these are unlikely to be true malignancies [57].

Studies on this topic are conflicting. A prospective within-patient study by Daneshmand et al. found a false positive rate of 9.1% for both BLC and WLC [41]. However, another prospective within-patient study with 61 patients found that the positive predictive value of BLC was 48%, compared to 79% with WLC (*p* < 0.05), with another similarly designed study of 45 patients yielding a 3–5% higher false positive rate for BLC (no *p*-value). Li et al. examined a prospective cohort of 128 patients and found a BLC false positive rate of 23.4% and WLC rate of 16.0% (no *p*-value) [58]. It is worth noting that in 2014, Witjes et al. published expert guidelines against the use of BLC to evaluate completeness of a previous resection, citing concerns regarding granulation tissue and healing contributing to fluorescence rather than a true residual lesion [59].

Some findings also highlight a relationship between false positives and sex, with one retrospective study finding higher false positive rates in females (35.9% vs. 28.5%, *p* = 0.008) [60]. Another retrospective study in 2009 showed that females were over twice as likely to have a false positive (*p* = 0.005) and sex was more associated with false positives than previous BCG instillations and previous radiotherapy, possibly explained by females having higher rates of urinary tract infections and associated urothelial inflammation, which can contribute to non-specific fluorescence on BLC [61, 62].

Physicians performing BLC can decrease risk of false positives and unnecessary resections by considering these scenarios and timing interventions wisely. Clinicians must be mindful of BCG-induced bladder irritation and increased HAL uptake beyond the immediate post-therapy period [39, 63]. The positive predictive value of BLC in patients with any history of recent BCG therapy may only be 60% of that in patients who are BCG naïve, some authors recommend waiting 9–12 weeks before BLC in the post BCG setting [63, 64].

Lastly, BLC experience is also an important factor. Gravas et al. showed that residents were able to achieve excellent consensus with experienced urologists after 30 cases [65]. The learning curve of BLC is not well-studied, but early interpretation of tumor versus confounding fluorescence may lead to more false positives and negatives.

### Muscle Invasive Bladder Cancer

BLC is not mentioned in the any current guidelines for muscle invasive bladder cancer [66]. Muscle invasive bladder cancer (MIBC) comprises 20–25% of all BC cases [67, 68]. Muscle invasive lesions often present with a larger papillary appearance but may have occult regions nearby that are missed on standard cystoscopy. In a retrospective analysis of 1257 patients of varying BC staging, Ahmadi et al. found that 6% of high-grade T2 lesions were detected only by BLC (no *p*-value) [69].

BLC may become useful in the MIBC space as a tool for selecting MIBC bladder preservation candidates. The ideal patient for bladder preservation has low volume T2 disease, absence of CIS, and maximal TURBT with regular surveillance [70]. BLC may prove a useful tool to verify low volume disease and could help facilitate maximal TURBT and effective surveillance for patients with MIBC who elect for bladder preservation. There is a paucity of high-quality studies investigating the use of BLC in MIBC for therapy selection or surveillance. Patients with T2 disease comprised only a small portion of the Ahmadi et al. study’s population, and currently, more advanced disease (> cT2) is typically diagnosed via physical exam, standard TURBT, and/or imaging.

### Cost Effectiveness

Administration of BLC requires specialized equipment which increases the immediate cost compared to WLC. The prospective randomized Heer et al. study showed that BLC was £876 more expensive per person in the U.K. over a 3-year follow-up (*p* = 0.591) [51]. Given the long-term financial burden of recurrent bladder cancer, BLC might provide a cost benefit in some patients due to increased detection and more complete tumor resection. Studies examining BLC economics in the US have varying findings. Garfield et al. modeled that BLC decreased the 5-year cost for patients by $5290 (no *p*-value) [71]. Another study by Williams et al. predicted that BLC was marginally more expensive than WLC over 2 years but identified 9 additional recurrences that could have been more costly if not detected (no *p*-value) [72]. Both studies utilized models simulating possible outcomes and treatments for BC patients using Medicare cost data and published peer-reviewed BC data. However, a subsequent study expanded on the Williams et al. model to a 5-year timeframe hypothesized that while BLC can extract value from preventing recurrences, the current Medicare reimbursement structure does not appropriately cover cystoscopies and disincentivizes community-based ambulatory centers from adopting BLC more routinely [73].

Outside of the US, many studies have been done into BLC economics. Estimates are consistent in stating that BLC has an increased upfront cost and may or may not pay off with future recurrences prevented [74–76]. An important caveat is that reimbursement and equipment charges vary by country and treatment setting. As more physicians become proficient in BLC and health systems evolve, the validity of these could change, and each health system must evaluate whether BLC is a worthwhile investment. These studies also operate on decision tree models, meaning the data is simulated based on past patients or published data and predicted recurrences and future costs. More prospective investigation is needed to determine whether BLC provides a long-term economic benefit.

### Safety, Patient Perspectives, Provider Perspectives, and Office Use

The adverse effects of cystoscopy, including hematuria, postoperative discomfort, and transient sexual dysfunction, are well known, and patients generally prefer flexible over rigid cystoscopy [77–79]. BLC requires an additional step with the instillation of HAL solution. While a theoretical allergy risk exists, the prospective randomized study by Heer et al. showed similar adverse event rates between BLC and WLC (no *p*-value) [51]. An analysis by Witjes et al. of 6 studies and post-market data from over 200,000 patients found no serious adverse events directly attributed to HAL and only 4 possible hypersensitivity reactions [80, 81].

HAL is also safe for repeat use. In 2022, Pohar et al. conducted a prospective within-patient study showing that patients with multiple HAL exposures, including those who previously received intravesical therapy, did not experience more adverse events than first-time patients (*p* = 0.76) [82]. Earlier studies similarly found repeated HAL exposure was not associated with significant adverse events [41, 80].

Patients report a general satisfaction with BLC. Smith et al. evaluated surveillance BLC in a prospective cohort and found decreased anxiety among patients with negative pathology and low post-cystoscopy pain (no *p*-value) [83]. Over 90% of patients found BLC worthwhile, with 76% of patients being willing to pay out of pocket for the procedure. While positive, willingness to pay may not reflect actual behavior, and the lack of a control group limits interpretation of the impact of additional waiting time compared to WLFC [84]. Because BC has some of the lowest treatment patient satisfaction and patient-reported outcomes are limited, more investigation needs to be done into how BLC affects patient opinions on overall BC treatment [85].

Given patient preferences for flexible cystoscopy, BLFC could have expanded into a valid office-based tool. Several BLFC studies investigated its benefits in office surveillance and diagnostic settings, but data on office adoption trends are lacking [30, 40, 53]. These studies were largely conducted at larger institutions, where staffing needs and reduced clinic throughput due to BLC logistics are smaller deterrents than in community-based practices. Karl Storz^™^ discontinued production of the BLFC equipment on March 23, 2023, and per personal correspondence, will halt service of existing units on October 31, 2026 due to undisclosed supply chain issues [86]. If BLFC becomes available in the future, more investigation can be done into its logistics, economics, and efficacy.

Regardless of cystoscope format, BLC visualization requires use of Karl Storz^™^ equipment, which often requires purchase of additional materials if current setups are not optimized. The cost concerns mentioned before may be difficult for many office practices to justify, and while Medicare in the US is increasingly accepting of BLC coverage, private insurance company policies can vary widely. With a per-dose cost of $1000-$1500, BLC has potential to increase the cost-burden of an already costly disease if not used appropriately.

From the provider perspective, BLC can provide value in increasing diagnostic confidence [87]. However, there is a possibility that when physicians toggle between BLC and WLC on cystoscopy, the process itself results in a more thorough cystoscopy that results in better detection and confidence as an artifact. Nonetheless, the notable direct and indirect accommodations BLC requires, along with ensuring patients are familiar with the special steps required in conjunction with false positive risks, are not worth the benefits for some providers.

### Patient Demographic and Socioeconomic Factors Influencing BLC Utilization

The clinical utilizations of BLC are just as important as the population of patients receiving it, and recent studies suggest that social determinants of health (SDOH) may influence its utilization. In a retrospective study of 2,122 patients in the US, Bhatt et al. found that patients receiving BLC were younger, more likely to be married, less socioeconomically disadvantaged, and more likely to have private insurance (*p* < 0.05) [88].

Ladi-Seyedian et al. also retrospectively found that BLC had a significantly increased sensitivity for detecting NMIBC compared to WLC in Asian and White populations (*p* < 0.001 for both) but no difference in Black and Hispanic groups (*p* = 0.13, 1.0, respectively) [28]. However, the interpretation is nuanced. The Black and Hispanic populations did not experience a significant improvement using BLC because sensitivity using WLC in those groups was already over 92%. Asian patients had over twice as many CIS lesions as Hispanic patients, which could explain BLC’s amplified benefit in that cohort. Separately, Das et al. retrospectively found that there was no difference in survival, recurrence, or progression after BLC between Black and White patients (*p* > 0.05) [48]. These results suggest that BLC is effective in groups with higher prevalences of occult lesions, but any conclusions about race should be interpreted within the context of other SDOH.

### Post-Image Processing and Use of Artificial Intelligence

Computer-assisted image processing techniques and artificial intelligence (AI) are increasingly being explored to enhance the diagnostic capabilities of BLC. Chang et al. developed a method to correct the characteristic green hue and fogginess in BLC images, improving their perceptual quality and aiding in more accurate interpretation [89]. Another study introduced a digital staining technique transforming WLC images into BLC-like images [90]. Additionally, various deep convolutional neural networks (CNNs) to classify BLC images have achieved high sensitivity in predicting malignancy, stage, and grade that could assist providers in making diagnostic determinations [91]. The implementation of AI or image processing has various possibilities. Whether these techniques will disproportionately improve BLC or WLC remains to be seen.

### Future Directions and Innovations

There are multiple ongoing clinical trials investigating BLC cost-effectiveness, learning curve, patient perspectives, and recurrence rates [92–96]. One trial in Italy aims to examine the non-inferiority of BLC compared to WLC in patients who meet the staging criteria for a repeat resection [96]. Studies investigating whether BLC can decrease the use of adjunct procedures provide insight into benefits of BLC beyond detection. Additionally, combining BLC with biomarkers is an emerging approach for risk stratification that could enable more personalized surveillance and identify patients for intensified or de-escalated follow-up.

BLC has also been used to identify upper tract urothelial carcinomas. A preliminary prospective trial demonstrated higher sensitivity than WLC for upper tract urothelial carcinoma using blue light ureterorenoscopy (*p* < 0.05) [97]. Additionally, one case report documents the diagnosis of an upper tract tumor using rigid BLC in a patient with an incompetent ureteropelvic junction [98]. Patients with BC are at increased of upper tract urothelial carcinoma, representing a potential useful extension upon the utility of BLC. There is also one reported case of metastatic melanoma of the bladder diagnosed with BLC [99]. This may point towards use of blue light as a method to tag malignant pathologies other than NMIBC.

Lastly, the benefits of BLC are predicated on its adoption. Physicians who are more likely to use BLC tend to practice at academic hospitals with large case volumes, while lower volume or non-academic practices may use it less frequently, suggesting a potential unmet need [100]. Advantages of BLC could be masked by data from high-volume academic physicians, and its role as an assistive tool for new physicians is under-investigated. Finally, there is a need for standardization of technique. Variability in instillation, sitting procedures, and performance of BLC across centers underscores the need for standardized protocols to ensure reproducible outcomes.

## Conclusion

Blue-light cystoscopy is a useful and safe adjunct to traditional cystoscopy for the diagnosis and management of NMIBC. BLC demonstrates a higher sensitivity for NMIBC lesions on initial diagnosis and surveillance, with conflicting data on whether it is associated with reduced recurrence or progression rates. Questions around cost-effectiveness, standardization of provider technique, logistics, and long-term outcomes must be answered to optimize the strengths of BLC.

## Key References

- Heer R, Lewis R, Vadiveloo T, Yu G, Mariappan P, Cresswell J, et al. A Randomized Trial of PHOTOdynamic Surgery in Non-Muscle-Invasive Bladder Cancer. NEJM Evid. 2022;1(10):EVIDoa2200092. 10.1056/EVIDoa2200092.
  - Large randomized trial examining recurrence, cost-effectiveness, and health-related quality of life after initial PDD-guided TURBT vs. WLC-guided TURBT in patients with suspected NMIBC.
- Ladi-Seyedian S-S, Ghoreifi A, Konety B, Pohar K, Holzbeierlein JM, Taylor J, et al. Racial Differences in the Detection Rate of Bladder Cancer Using Blue Light Cystoscopy: Insights from a Multicenter Registry. Cancers. 2024;16(7):1268. 10.3390/cancers16071268.
  - Analysis of Cysview® prospective registry comparing BLC sensitivity among different racial groups.
- Miyake M, Nishimura N, Nakahama T, Nishimoto K, Oyama M, Matsushita Y, et al. Additional oncological benefit of photodynamic diagnosis with blue light cystoscopy in transurethral resection for primary non-muscle-invasive bladder cancer: A comparative study from experienced institutes. BJUI Compass. 2023;4(3):305-13. 10.1002/bco2.215.
  - Large retrospective cohort study comparing IBCG-defined progression and recurrence in patients post-BLC vs. post-WLC.
- Sari Motlagh R, Ghoreifi A, Yanagisawa T, Kawada T, Ahyai S, Merseburger AS, et al. Surveillance of non-muscle-invasive bladder cancer with blue-light cystoscopy: a meta-analysis. BJU Int. 2024;134(4):526-33. 10.1111/bju.16364.
  - A meta-analysis of 10 studies examining BLC vs. WLC detection of NMIBC recurrence in a surveillance setting.

## Data Availability

No datasets were generated or analysed during the current study.
